# Participation and relative cost of attendance by direct‐mail compared to opt‐in invitation strategy for HPV self‐sampling targeting cervical screening non‐attenders: A large‐scale, randomized, pragmatic study

**DOI:** 10.1002/ijc.35263

**Published:** 2024-11-23

**Authors:** Birgitte Tønnes Pedersen, Si Brask Sonne, Helle Pedersen, Emilie Korsgaard Andreasen, Reza Serizawa, Ditte Møller Ejegod, Jesper Bonde

**Affiliations:** ^1^ Department of Pathology Copenhagen University Hospital AHH‐Hvidovre Hvidovre Denmark

**Keywords:** cervical cancer prevention, cervical cancer screening, HPV, HPV self‐sampling

## Abstract

Broad accessibility to cervical cancer screening and high participation rate is essential to reduce cervical cancer incidence. HPV self‐sampling is an alternative to clinician collected cervical samples to increase accessibility and screening coverage. To inform on deployment strategies of HPV self‐sampling, this large‐scale, randomized, pragmatic study compared two invitation modalities; direct‐mail and opt‐in. The study included screening non‐attenders from the Capital Region of Denmark randomly allocated (1:4) to a direct‐mail or opt‐in invitation for cervical screening by HPV self‐sampling. Primary endpoint was screening participation; secondary endpoints were HPV prevalence and histology outcome. Adherence to follow‐up and cost were also evaluated. After exclusion of hysterectomized/non‐accessible women, 49,393 women were invited: 9639 by direct‐mail, and 39,754 by the opt‐in offer. A direct‐mail invitation for HPV self‐sampling yielded a significant higher participation than an opt‐in invitation. HPV self‐sample participation for direct‐mail was 25.2% (*n* = 2426), opt‐in participation was 20.2% (*n* = 8047), adjusted OR = 1.27, 95% CI 1.20–1.34. Participation increased with age (*p* < .0001) for both strategies and decreased with screening history of non‐attendance (*p* < .0001). Interaction between invitation strategy and age/screening history was found; more women below 50 years of age participated by direct‐mail compared to opt‐in (*p* < .0001) and higher participation by direct‐mail group was found in women with a short history of non‐attendance (*p* < .0001). Participation of long‐term unscreened women was similar between arms. The relative cost was ≈14 HPV self‐sample kits distributed per additional participant by direct‐mail over opt‐in. HPV prevalence, adherence to follow‐up, and detection of high‐grade cervical intraepithelial neoplasia was similar between invitation strategies.

## Graphical abstract

What's new?

Human papillomavirus (HPV) self‐sampling increases cervical screening coverage. However, self‐sampling kits may be directly mailed to all women or have to be actively ordered, with a potential impact on the resource requirements vs participation and disease detection. This study shows that, in a real‐life organized screening setting, the opt‐in offer performs better than previously reported. Compared to an opt‐in invitation, the direct‐mail invitation yields a significantly higher participation, with a relative cost of 14 HPV self‐sampling kits distributed per additional direct‐mail participant. No difference in the detection of high‐grade cervical intraepithelial neoplasia was found between the two invitation strategies.

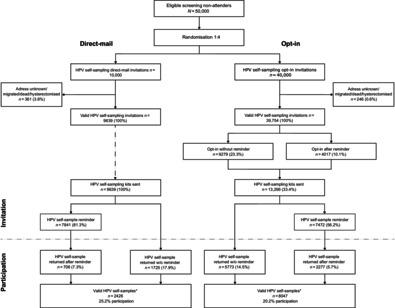

## 1 INTRODUCTION

Cervical cancer is the fourth most common cancer in women worldwide, it has an early onset and is one of the most frequent cancers in women younger than 45 years.^1^ Cervical cancer is predominantly the result of a persistent infection with carcinogenic human papillomavirus (HPV) genotypes^2^ and is preventable through vaccination, screening, and treatment of precursor lesions.^3^ Countries with organized screening programs with high population coverage has reduced cervical cancer incidence and mortality dramatically^4^ and screening is a key pillar in the World Health Organization strategy to eliminate cervical cancer.^5^ A successful cervical screening effort requires broad accessibility to, and participation by, eligible women. Non‐participation is a major determinant for cervical cancer risk^6^ and a previous register‐based study showed that 45% of patients diagnosed with cervical cancer were screening non‐participants.^7^ In Denmark, 5‐year screening coverage is currently 74%, however screening participation is wavering.^8^ Even the best attended programs struggle to obtain a 5‐year coverage exceeding 75%.^9^ Reasons for non‐participation are multifaceted. They include reluctance to undergo pelvic examination for a non‐acute condition, socioeconomic background, lack of awareness, cultural non‐acceptance of benign gynecological examinations, and practical and emotional barriers.^10^, ^11^, ^12^ Cervical screening has relied on clinician collected cytology samples^13^ for decades, but cytology is increasingly substituted for molecular HPV screening as methodology‐of‐choice given the superior negative predictive value and clinical sensitivity of HPV screening over cytology‐based strategies.^14^, ^15^ With the increasing implementation of HPV technology, HPV self‐sampling has emerged as a compelling supplement to clinician collected screening samples to increase screening participation. An HPV self‐sample is collected without the pelvic exam and by the woman herself at her convenience. Also, self‐sampling can be done in the privacy of own home or assisted in a health care setting. HPV self‐sampling thereby mitigates several of the known barriers to participation in screening and offering self‐samples to women has proven to increase cervical screening participation^16^, ^17^ regardless of whether the initiatives targets screening non‐attenders^18^, ^19^, ^20^, ^21^ specific age‐groups^22^ or other sub‐demographics of women.^23^ Today, countries like the Netherlands, Sweden, Australia, and Malaysia offer HPV self‐sampling as a primary screening approach, whereas Denmark systematically offers HPV self‐sampling to under‐screened women.^24^ A key feature of HPV self‐sampling is distribution by mail, pharmacies, or similar outlets directly to the woman thereby removing the need for a clinical appointment. Diagnostic analysis is conducted upon return of the sample to a laboratory. For mail‐based initiatives, two invitation strategies dominate; *direct‐mail* (also called *mail‐to‐all* or *send‐to‐all*) where women are mailed an HPV self‐sampling kit directly to their home address together with the invitation to participate in screening, or *opt‐in* where women are invited to screening but required to actively order the HPV self‐sampling kit. The recent meta‐analysis on randomized controlled trials by Costa et al.^16^ decisively shows, that when evaluated as intention‐to‐treat, both invitation strategies have proven more effective to increase screening participation than repeated, or dedicated invitations/reminders to have a regular clinician collected screening sample. In general, direct‐mail generates the largest participation compared to opt‐in, however the difference depends on the specific local context and set‐up.^16^ From a logistic point‐of‐view, direct‐mail generates a significant higher rate of kits not used, compared to opt‐in where only women who actively request the kit receives it. Having said that, not all women who opt‐in returns the kit for analysis.^20^ To date, no studies have assessed the difference between the direct‐mail and opt‐in strategies in terms of resource requirements vs. participation and disease detection.^16^ The Capital Region of Denmark covers approximately 1/3 of the Danish cervical cancer screening population. HPV self‐sampling is routinely offered 6 months after a regular screening invitation if no screening sample has been registered in the national database. It is additionally offered with a dedicated HPV self‐sampling invitation to women who are long‐term screening non‐attenders. Cervical cancer screening and HPV self‐sampling is free‐of‐charge under the regional screening program. For both HPV self‐sampling indications, the opt‐in invitation is the current standard of care in the Capital region of Denmark. This choice of strategy was originally chosen following the successful 2014–2015 pilot implementation study among screening non‐attenders.^25^, ^26^, ^27^ However, based on the results of the current meta‐analysis,^16^ a smaller scale Danish study,^28^ and as part of our quality improvement efforts in the region, we wanted to investigate if a direct‐mail approach would be a better strategy than opt‐in. We therefore conducted a large‐scale pragmatic, randomized study embedded in the cervical screening program of the Capital Region of Denmark targeting screening non‐attenders to assess and compare screening participation for direct‐mail vs. opt‐in invitation strategies. Furthermore, we evaluated the detection of high‐grade cervical intraepithelial neoplasia (CIN) and the cost of the two approaches.

## 2 MATERIALS AND METHODS

### 2.1 Study design and participants

This study had a pragmatic, randomized, controlled design, embedded into the public health care HPV self‐sampling service, targeting cervical cancer screening non‐attenders in the Capital Region of Denmark. Screening non‐attenders were defined as women with no screening sample registered for >4 years (age 27–50 years) or >6 years (age 51–64 years).^20^ Women eligible for HPV self‐sampling according to the criterion were identified in the Danish National Pathology Database (Patobank) and randomly assigned to two groups: the *direct‐mail* group (intervention, *n* = 10,000) or the *opt‐in* group (control, *n* = 40,000). The primary endpoint of the study was screening participation, secondary endpoints HPV prevalence, and histology outcome. Adherence to follow‐up and relative cost were evaluated. The overall sample size of the two groups was the operational target of 50,000 invited women for HPV self‐sampling set by the Capital Region of Denmark. Based on experiences from previous Danish studies^27^, ^28^ and the cost of changing a part of the current program from opt‐in to direct‐mail, the target group was pragmatically divided proportionally as 1:4 for the direct‐mail and opt‐in group, respectively. Women in the direct‐mail group received an invitation to participate along with an HPV self‐sampling kit delivered directly to their home address. The women in the opt‐in group received an invitation by national digital mail, with the option to order the HPV self‐sampling kit through either the HPV self‐sampling website of the Capital Region of Denmark (www.HPV-hjemmetest.dk), dedicated service phone, or dedicated e‐mail recipient, as previously described.^20^ Upon active acceptance, women from the opt‐in group received an HPV self‐sampling kit by mail. Reminders were issued if no response was recorded 8 weeks after issuing the HPV self‐sampling invitation. For both arms, women who received an HPV self‐sampling kit, but did not return the sample within 8 weeks, were automatically issued a reminder to return the test for analysis. No time limitation was enforced between sampling and return of sample for laboratory analysis, however only samples received up until 31st December 2021 were included in the study. If an HPV self‐sample kit was lost/damaged or the HPV self‐sample had an invalid result, replacement kits were issued to women. The HPV self‐sample kit was identical for the two strategy groups and represents the standard‐of‐care HPV self‐sample kit including an Evalyn™ brush offered by the Capital Region of Denmark. Prior to shipment, the included Evalyn™ brush was electronically linked to the woman by a unique RFID chip as previously described.^27^ The HPV self‐sampling kit was mailed to the women in the direct‐mail group from June 2020 to August 2020 and the opt‐in offer was provided to the women in the opt‐in group from September 2020 to January 2021. The reporting of the study was planned according to the extended CONSORT statement.^29^

### 2.2 Data sources and definitions

**FIGURE 1:**
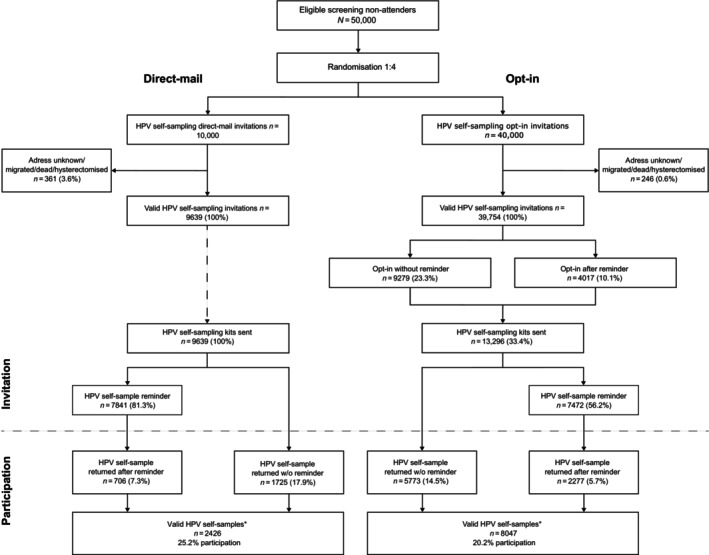
Study profile. *In total, 8 women did not return a valid HPV self‐sample after an invalid result: 5 in the direct‐mail group and 3 in the opt‐in group.

HPV self‐sample participation was defined as a valid HPV self‐sample returned to the laboratory (Figure 1). In this study, all HPV self‐samples returned up until 31st December 2021 were included. Additionally, we evaluated the Intention‐To‐Screen (ITS) participation, defined as participation with either an HPV self‐sample or a clinician collected screening sample. The latter was defined as a registered screening sample within 12 months after the HPV self‐sampling invitation, excluding samples registered with an ordinary screening invitation. Data was retrieved from the HPV self‐sample software database (The Capital Region of Denmark), including date of shipment, invitation reminder, return‐of‐kit reminder, replacement kit (if any), and return‐of‐kit date. Data on clinician collected screening samples, and the result of the HPV self‐sample and potential follow‐up cytology/histology outcome were retrieved from Patobank in July 2023. The follow‐up period from a positive HPV self‐sample was 18 months for all women to allow for (i) a clinician collected co‐test following an HPV‐positive self‐sample (recommended within 3 months after index HPV self‐sample, reminder issued to women after 3 months), (ii) subsequent 2nd co‐test if any (within 12 months of index sample) (iii) and/or referral for colposcopy (within 3 months after positive clinician collected follow‐up sample) (Figure S1). Adherence to a clinician collected follow‐up sample after HPV‐positive index sample was evaluated according to Figure S1. Screening history was retrieved from the Patobank and divided into three categories: (i) Missed one screening round; Last sample registered 4–5 years ago for women below 50, or last sample registered 6–7 years ago for women above 50; (ii) Missed more than one screening round; Last sample registered 5–10 years ago for women below 50, and 7–12 years ago for women above 50 years, and (iii) Long‐ term unscreened; Last sample registered more than 10 years ago for women below 50 and more than 12 years ago for women above 50. Histology outcomes were classified according to the CIN grading system and reported in aggregated groups defined as ≤CIN1, ≥CIN2 (including CIN not otherwise specified) and ≥CIN3. If multiple histology samples were registered within the follow‐up period of the study after a positive HPV self‐sample, the worst‐case histology was designated as outcome. Histology outcomes were summarized for women who adhered completely to the triage algorithm and for all women with a positive HPV self‐sample and a subsequent biopsy regardless of adherence to screening algorithm (Figure S1).

### 2.3 Sample processing and HPV analysis

HPV self‐samples returned to the laboratory were linked to the woman using the RFID chip to ensure secure patient ID. Laboratory processing of the HPV self‐samples, regardless of invitation strategy group, was conducted using the BD Onclarity HPV test as described in detail previously by Ejegod et al.^20^

### 2.4 Statistical analysis

Data for the two invitation strategies are reported as number and proportion with a corresponding 95% confidence interval (CI) in total and by age‐ and screening history‐group. The participation rate for the two offers was calculated based on the number of invited women, however women who were excluded from the study were not included in the denominator (Figure 1). Time from dispatching of kit to recipient until reception of the returned HPV self‐sample at the laboratory are reported as median and interquartile range (IQR). Prevalence of HPV was calculated as % of high‐risk (HR) HPV‐positive self‐samples out of total number of returned HPV self‐samples. Time from positive HPV self‐sample to clinician collected follow‐up sample was analyzed by non‐parametric median test. A logistic regression model was applied to describe the association between invitation strategy and participation including all invited women. HPV prevalence for the two groups was evaluated by logistic regression including all HPV self‐sample participants in the model. The association between invitation strategy and detection of high‐grade CIN, defined as ≥CIN2 and ≥CIN3 in all women with a positive HPV self‐sample and a histology sample, regardless of adherence to screening algorithm, were analyzed by logistic regression as well. For comparison of invitation strategies, opt‐in was designated as standard‐of‐care. All models were adjusted by age and screening history as explanatory variables. Adjusted and crude odds‐ratio (OR) including 95% CI were estimated. Statistical analysis was performed using SAS v 9.4 (SAS Institute Inc., Cary, NC, USA).

### 2.5 Cost calculation

As price structures differ between regions and countries, a generic approach was applied where cost of attender was measured in number of HPV self‐sample kits sent. The relative cost of direct‐mail vs. opt‐in in terms of participation was calculated as the difference in number of kits sent per additional woman participating. The cost of detection of ≥CIN2 and ≥CIN3 was calculated as number of kits for detection of one case of high‐grade CIN by direct‐mail divided by number of kits for detection of one case of high‐grade CIN by opt‐in. Number of kits included replacement kits.

## 3 RESULTS

### 3.1 Participation

**FIGURE 2:**
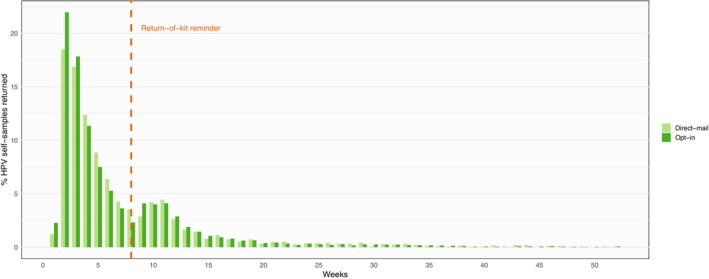
Time to return of HPV self‐sample. Data shows received HPV self‐samples in % of total number of HPV self‐samples returned within a year after shipment. The distribution is shown over time (in weeks) calculated from date of dispatching of kit to recipient until date of reception of the returned HPV self‐sample in the laboratory.

A total of 49,393 women were included in the study: 9639 women in the direct‐mail group, and 39,754 in the opt‐in group. Exclusions (*n* = 607) consisted of hysterectomized women (self‐reported), undeliverable/unknown at the address, or migrated (Figure 1). All 9639 women in the direct‐mail group received an HPV self‐sampling kit directly. For opt‐in, 33.4% (*n* = 13,296) accepted the offer and received an HPV self‐sampling kit. A limited number of women in the direct‐mail group (*n* = 84) and in the opt‐in group (*n* = 230) received a replacement kit upon request by the woman (*n* = 76 for direct‐mail and *n* = 216 for opt‐in), or after an invalid HPV self‐sample (*n* = 8 for direct‐mail and *n* = 14 for opt‐in). The resulting HPV self‐sample participation for direct‐mail was 25.2%, (95% CI 24.3–26.0, *n* = 2426), whereas participation in the opt‐in group was 20.2% (95% CI 19.8–20.6, *n* = 8047, Table 1). The direct‐mail participation was significantly higher than opt‐in participation, OR = 1.33 (95% CI 1.26–1.40, *p* < .0001). Participation increased with age (*p* < .0001) for both strategies and decreased with time since last screening (*p* < .0001) (Table 1). After adjustment, participation in the direct‐mail group remained significantly increased compared to opt‐in (adjusted OR = 1.27, 95% CI 1.20–1.34, *p* < .0001). The analysis showed an interaction between the invitation strategy and age, more women in the age‐groups below 50 years participated when the HPV self‐sample kit was directly mailed compared to opt‐in (27–29 years: OR = 1.51, 95% CI 1.31–1.74, *p* < .0001; 30–39 years: OR = 1.41, 95% CI 1.28–1.55, *p* < .0001; 40–49 years: OR = 1.26, 95% CI 1.14–1.40, *p* < .0001). No significant difference in participation according to invitation strategy was found in women above 50 years of age (50–59 years: OR = 1.11, 95% CI 0.98–1.25; 60–65 years: OR = 0.94, 95% CI 0.80–1.10). Furthermore, an interaction between invitation strategy and time since last screening was found, with higher participation in the direct‐mail group compared to the opt‐in group for unscreened women who missed one (OR = 1.42, 95% CI 1.29–1.55, *p* < .0001) or more than one screening round (OR = 1.39, 95% CI 1.28–1.50, *p* < .0001), whereas no difference between strategies was observed among long‐term unscreened women (OR = 0.91, 95% CI 0.81–1.02, *p* = .1038). The median (IQR) time from shipment of HPV self‐sample kit to return of sample to laboratory was 30 (47) and 28 (47) days for direct‐mail and opt‐in, respectively. Independent of strategy, return of HPV self‐samples peaked 2 weeks after shipment, then decreased until a reminder was sent 8 weeks after shipment, resulting in a second peak of returned HPV self‐samples (Figure 2). A subset of the women chose to be screened by a clinician collected sample after receiving invitation for HPV self‐sampling. This added 977 (10.1%, 95% CI 9.5–10.8) women screened in the direct‐mail group, and 4316 (10.9%, 95% CI 10.6–11.2) women screened in the opt‐in group resulting in a total ITS participation of 35.3% (95% CI 34.3–36.3) in the direct‐mail group and 31.1% (95% CI 30.6–31.6) in the opt‐in group (Table 1).

**TABLE 1:** HPV self‐sampling and overall screening participation (ITS) by invitation strategy.

Age (years)	Direct‐mail			Opt‐in		
	Invited^a^	HPV self‐sample participants^b^	ITS participants^b^	Invited^a^	HPV self‐sample participants^b^	ITS participants^b^
	*n*	*n*	*n*	*n*	*n*	*n*
	%	%	%	%	%	%
	[95% CI]	[95% CI]	[95% CI]	[95% CI]	[95% CI]	[95% CI]
All women	9639	2426	3403	39,754	8047	12,363
	100.0%	25.2%	35.3%	100.0%	20.2%	31.1%
	–	[24.3–26.0]	[34.3–36.3]	–	[19.8–20.6]	[30.6–31.6]
27–29	1478	326	518	7024	1105	2051
	15.3%	22.1%	35.0%	17.7%	15.7%	29.2%
	[14.6–16.1]	[20.0–24.3]	[32.6–37.5]	[17.3–18.0]	[14.9–16.6]	[28.1–30.3]
30–39	2975	714	1096	12,256	2181	3883
	30.9%	24.0%	36.8%	30.8%	17.8%	31.7%
	[29.9–31.8]	[22.5–25.6]	[35.1–38.6]	[30.4–31.3]	[17.1–18.5]	[30.9–32.5]
40–49	2467	686	944	9434	2109	3181
	25.6%	27.8%	38.3%	23.7%	22.4%	33.7%
	[24.7–26.5]	[26.0–29.6]	[36.3–40.2]	[23.3–24.2]	[21.5–23.2]	[32.8–34.7]
50–59	1689	442	550	6774	1554	1985
	17.5%	26.2%	32.6%	17.0%	22.9%	29.3%
	[16.8–18.3]	[24.1–28.3]	[30.3–34.9]	[16.7–17.4]	[21.9–24.0]	[28.2–30.4]
60–65	1030	258	295	4266	1098	1263
	10.7%	25.0%	28.6	10.7%	25.7%	29.6%
	[10.1–11.3]	[22.4–27.8]	[25.9–31.5]	[10.4–11.0]	[24.4–27.1]	[28.2–31.0]
**Screening history**						
Missed one screening round	2941	892	1404	10,049	2318	3970
	30.5%	30.3%	47.7%	25.3%	23.1%	39.5%
	[29.6–31.4]	[28.7–32.0]	[45.9–49.6]	[24.9–25.7]	[22.2–23.9]	[38.5–40.5]
Missed more than one screening round	4190	1130	1488	16,683	3463	5127
	43.5%	27.0%	35.5%	42.0%	20.8%	30.7%
	[42.5–44.5]	[25.6–28.3]	[34.1–37.0]	[41.5–42.5]	[20.1–21.4]	[30.0–31.4]
Long‐term unscreened	2508	404	511	13,022	2266	3266
	26.0%	16.1%	20.4%	32.8%	17.4%	25.1%
	[25.1–26.9]	[14.7–17.6]	[18.8–22.0]	[32.3–33.2]	[16.8–18.1]	[24.3–25.8]

Abbreviations: CI, confidence interval; ITS, intention‐to‐screen (HPV self‐sample and clinician collected screening sample in total).

^a^ Presents number and column % [95% CI]. Denominator: number of invited women in invitation strategy group.

^b^ Presents number and row % [95% CI]. Denominator: number of women within in the respective age‐ and screening history group.

### 3.2 HPV prevalence

HPV prevalence was similar across the two groups: 14.6% (95% CI 13.2–16.0) vs. 14.6% (95% CI 13.9–15.4) for direct‐mail and opt‐in, respectively (OR = 0.98, 95% CI 0.86–1.12). For both groups, the HPV prevalence was highest in women aged 27–29 years and decreased with increasing age (*p* < .0001). No specific pattern between HPV prevalence, screening history, and invitation strategy was found (Table 2). When adjusting for age, no difference in HPV prevalence between the two strategies was observed (adjusted OR = 0.96, 95% CI 0.85–1.10).

**TABLE 2:** HPV prevalence and adherence to follow‐up according to invitation strategy.

Age (years)	Direct‐mail			Opt‐in		
	HPV prevalence self‐sample^a^	Adherence to follow‐up^b^	HPV prevalence clinician collected follow‐up sample^c^	HPV prevalence self‐sample^a^	Adherence to follow‐ up^b^	HPV prevalence clinician collected follow‐up sample^c^
	*n*	*n*	*n*	*n*	*n*	*n*
	%	%	%	%	%	%
	[95% CI]	[95% CI]	[95% CI]	[95% CI]	[95% CI]	[95% CI]
All women	353	305	186	1178	1056	634
	14.6%	86.4%	61.0%	14.6%	89.6%	60.0%
	[13.2–16.0]	[82.4–89.8]	[55.3–66.5]	[13.9–15.3]	[87.8–91.3]	[57.0–63.0]
27–29	83	69	43	262	239	172
	25.5%	83.1%	62.3%	23.7%	91.2%	72.0%
	[20.8–30.6]	[73.3–90.5]	[49.8–73.7]	[21.2–26.3]	[87.1–94.4]	[65.8–77.6]
30–39	112	101	70	374	334	222
	15.7%	90.2%	69.3%	17.1%	89.3%	66.5%
	[13.1–18.6]	[83.1–95.0]	[59.3–78.1]	[15.6–18.8]	[85.7–92.2]	[61.1–71.5]
40–49	80	68	41	254	228	133
	11.7%	85.0%	60.3%	12.0%	89.8%	58.3%
	[9.4–14.3]	[75.3–92.0]	[47.7–72]	[10.7–13.5]	[85.4–93.2]	[51.6–64.8]
50–59	52	45	25	156	138	61
	11.8%	86.5%	55.6%	10.0%	88.5%	44.2%
	[8.9–15.1]	[74.2–94.4]	[40.0–70.4]	[8.6–11.6]	[82.4–93.0]	[35.8–52.9]
60–65	26	22	7	132	117	46
	10.1%	84.6%	31.8%	12.0%	88.6%	39.3%
	[6.7–14.4]	[65.1–95.6]	[13.9–54.9]	[10.2–14.1]	[82.0–93.5]	[30.4–48.8]
**Screening history**						
Missed one screening round	120	104	60	358	329	202
	13.5%	86.7%	57.7%	15.4%	91.9%	61.4%
	[11.3–15.9]	[79.3–92.2]	[47.6–67.3]	[14.0–17.0]	[88.6–94.5]	[55.9–66.7]
Missed more than one screening round	189	164	101	536	478	282
	16.7%	86.8%	61.6%	15.5%	89.2%	59.0%
	[14.6–19.0]	[81.1–91.3]	[53.7–69.1]	[14.3–16.7]	[86.2–91.7]	[54.4–63.4]
Long‐term unscreened	44	37	25	284	249	150
	10,9%	84.1%	67.6%	12.5%	87.7%	60.2%
	[8.0–14.3]	[69.9–93.4]	[50.2–82.0]	[11.2–14.0]	[83.3–91.3]	[53.9–66.4]

Abbreviation: CI, confidence interval.

^a^ Presents number and % [95% CI] of women with a positive HPV self‐sample out of valid HPV self‐samples received within age, screening history, and invitation strategy group.

^b^ Presents number and % [95% CI] of women adhering to recommended follow‐up (clinician collected follow‐up sample) out of HPV‐positive self‐samples received within age, screening history, and invitation strategy group.

^c^ Presents number and % [95% CI] of women with an HPV‐positive clinician collected follow‐up sample out of clinician collected follow‐up samples received within age, screening history, and invitation strategy group.

### 3.3 Adherence to follow‐up recommendations

Following a positive HPV self‐sample, adherence to the recommended clinician collected follow‐up sample was found to be similar across the groups; 86.4% for direct‐mail (95% CI 82.4–89.8) vs. 89.6% for opt‐ in (95% CI 87.8–91.3). Women in the opt‐in group went slightly faster to get a follow‐up sample after a positive HPV self‐sample than women in the direct‐mail group (direct‐mail: 33 days vs. opt‐in: 28 days, *p* = .0293). HPV prevalence in the follow‐up samples was similar in the two groups: 61.0% (95% CI 55.3–66.5) for direct‐mail vs. 60.0% (95% CI 57.0–63.0) for opt‐in (Table 2). Clinical management of clinician collected follow‐up samples is found in Figure S1. With respect to follow‐up triage outcome, we found no difference between the direct‐mail and opt‐in groups.

### 3.4 Histology outcome

Overall, 3.0% (95% CI 2.4–3.8, *n* = 73) of the returned HPV self‐samples in the direct‐mail group and 3.7% (95% CI 3.3–4.1, *n* = 295) in the opt‐in group resulted in colposcopy and biopsies (Table 3). A small subset of the women (*n* = 7 for direct‐mail, *n* = 34 for opt‐in) went directly for colposcopy following a positive HPV self‐sample. The proportion of women with a detected ≥CIN2 out of those who returned an HPV self‐sample was similar between groups; 1.4% (95% CI 0.9–1.9, *n* = 33) for direct‐mail and 1.6% (95% CI 1.3–1.9, *n* = 129) for opt‐in. Likewise, the proportion of ≥CIN3 was similar; 0.9% (95% CI 0.6–1.4, *n* = 22) for direct‐mail and 1.1% (95% CI 0.9–1.3, *n* = 86) for the opt‐in group (Table 3). When comparing the two strategies for detection of high‐grade CIN, including all valid HPV self‐samples, and adjusting for age and screening history, the logistic regression model revealed no difference between direct‐mail and opt‐in for detection of ≥CIN2 (adjusted OR = 0.84, 95% CI 0.57–1.23) or ≥CIN3 (adjusted OR = 0.86, 95% CI 0.54–1.38). However, in the pooled population (data not shown), age was associated with both ≥CIN2 and ≥CIN3 detection (*p* < .0001). Women of 50 years or older had lower risk of ≥CIN2 and ≥CIN3 compared to participants below age 50. Screening history was significantly associated with ≥CIN3 detection, with long‐term unscreened women at higher risk of ≥CIN3 (*p* = .0330). One and three cancer cases were detected in the direct‐mail group and opt‐in group, respectively.

**TABLE 3:** Histology outcome by age group and screening history according to invitation strategy.

Age (years)	Direct‐mail					Opt‐in				
	Histology in women with HPV+ self‐sample adherent to screening algorithm	Histology in women with HPV+ self‐sample and subsequent biopsy	≤CIN1	≥CIN2	≥CIN3	Histology in women with HPV+ self‐sample adherent to screening algorithm	Histology in women with HPV+ self‐sample and subsequent biopsy	≤CIN1	≥CIN2	≥CIN3
	*n*	*n*	*n*	*n*	*n*	*n*	*n*	*n*	*n*	*n*
	%	%	%	%	%	%	%	%	%	%
	[95% CI]	[95% CI]	[95% CI]	[95% CI]	[95% CI]	[95% CI]	[95% CI]	[95% CI]	[95% CI]	[95% CI]
All	66	73	40	33	22	261	295	166	129	86
	2.7%	3.0%	1.6%	1.4%	0.9%	3.2%	3.7%	2.1%	1.6%	1.1%
	[2.1–3.4]	[2.4–3.8]	[1.2–2.2]	[0.9–1.9]	[0.6–1.4]	[2.9–3.7]	[3.3–4.1]	[1.8–2.4]	[1.3–1.9]	[0.9–1.3]
27–29	16	16	9	7	5	72	76	45	31	19
	4.9%	4.9%	2.8%	2.1%	1.5%	6.5%	6.9%	4.1	2.8%	1.7%
	[2.8–7.8]	[2.8–7.8]	[1.3–5.2]	[0.9–4.4]	[0.5–3.5]	[5.1–8.1]	[5.5–8.5]	[3.0–5.4]	[1.9–4.0]	[1.0–2.7]
30–39	24	26	16	10	7	75	85	40	45	27
	3.4%	3.6%	2.2%	1.4%	1.0%	3.4%	3.9%	1.8	2.1%	1.2%
	[2.2–5.0]	[2.4–5.38]	[1.3–3.6]	[0.7–2.6]	[0.4–1.0]	[2.7–4.3]	[3.1–4.8]	[1.3–2.5]	[1.5–2.8]	[0.8–1.8]
40–49	13	15	6	9	7	68	78	40	38	31
	1.9%	2.2%	0.9%	1.3%	1.0%	3.2%	3.7%	1.9	1.8%	1.5%
	[1.0–3.2]	[1.2–3.6]	[0.3–1.9]	[0.6–2.5]	[0.4–1.0]	[2.5–4.1]	[2.9–4.6]	[1.4–2.6]	[1.3–2.5]	[1.0–2.1]
50–59	11	13	7	6	3	27	33	25	8	5
	2.5%	2.9%	1.6%	1.4%	0.7%	1.7%	2.1%	1.6	0.5%	0.3%
	[1.2–4.4]	[1.6–5.0]	[0.6–3.2]	[0.5–2.9]	[0.1–2.0]	[1.1–2.5]	[1.5–3.0]	[1.0–2.4]	[0.2–1.0]	[0.1–0.7]
60–65	2	3	2	1	0	19	23	16	7	4
	0.8%	1.2%	0.8%	0.4%	0.0%	1.7%	2.1%	1.5	0.6%	0.4%
	[0.1–2.8]	[0.2–3.4]	[0.1–2.8]	[0.0–2.1]	[−]	[1.0–2.7]	[1.3–3.1]	[0.8–2.4]	[0.3–1.3]	[0.1–0.9]
**Screening history**										
Missed one screening round	30	33	20	13	9	84	92	56	36	20
	3.4%	3.7%	2.2%	1.5%	1.0%	3.6%	4.0%	2.4%	1.6%	0.9%
	[2.3–4.8]	[2.6–5.2]	[1.4–3.4]	[0.8–2.5]	[0.5–1.0]	[2.9–4.5]	[3.2–4.5]	[1.8–3.1]	[1.1–2.1]	[0.5–1.0]
Missed more than one screening round	27	31	16	15	10	118	133	78	55	34
	2.4%	2.7%	1.4%	1.3%	0.9%	3.4%	3.8%	2.3%	1.6%	1.0%
	[1.6–3.5]	[1.9–3.9]	[1.8–3.1]	[0.7–2.2]	[0.4–1.6]	[2.8–4.1]	[3.2–4.5]	[1.8–2.8]	[1.2–2.1]	[0.7–1.0]
Long‐term un‐screened	9	9	4	5	3	59	70	32	38	32
	2.2%	2.2%	1.0%	1.2%	0.7%	2.6%	3.1%	1.4%	1.7%	1.4%
	[1.0–4.2]	[1.0–4.2]	[0.8–2.3]	[0.4–2.9]	[0.2–2.2]	[2.0–3.3]	[2.4–3.9]	[1.0–2.0]	[1.2–2.3]	[1.0–2.0]

*Note*: Presents number and % [95% CI] of biopsies and histology outcome of women in the respective age‐, screening history, and invitation strategy group. Denominator: number of returned HPV self‐samples within age, screening history, and invitation strategy group.

Abbreviations: CI, confidence interval; CIN, cervical intraepithelial neoplasia.

### 3.5 Cost of invitation strategies

In the direct‐mail group, 9723 HPV self‐sample kits were mailed to 9639 women and resulted in a total of 2426 valid screening samples, corresponding to 25.0% (95% CI 24.1–25.8) of the total amount of mailed HPV self‐sample kits and 25.2% (95% CI 24.3–26.0) participation. In the opt‐in group, 13,526 HPV self‐sample kits were distributed after an invitation to 39,754 women, corresponding to 34.0% (95% CI 33.6–34.5) HPV self‐sample kits mailed per invitation. This resulted in 8047 valid screening samples, corresponding to 59.5% (95% CI 58.8–60.3) of the shipped HPV self‐sample kits and a participation of 20.2% (95%CI 19.8–20.6). The ratio of HPV self‐sample kits sent compared to valid screening samples received in the laboratory was 4.0 for the direct‐mail group compared to 1.7 in the opt‐in group, resulting in a direct‐mail to opt‐in ratio of 2.4 kits per women screened. Using the opt‐in group as reference, the relative cost per additional woman screened in the direct‐mail group amounted to ≈14 HPV self‐sample kits distributed per extra woman screened. With respect to detected ≥CIN2 cases, 33 cases corresponding to 0.34% of invited woman (95% CI 0.20–0.50) and 0.34% of mailed HPV self‐sample kits (95% CI 0.20–0.50) were detected in the direct‐mail. In the opt‐in group, 129 cases of ≥CIN2 corresponding to 0.32% per invited woman, (95% CI 0.30–0.40) and 0.95% per distributed HPV self‐sample kit (95% CI 0.80–1.10) were detected. For detection of ≥CIN3 cases, 22 cases corresponding to 0.23% per invited woman (95% CI 0.10–0.30) and 0.23% per mailed HPV self‐sample kit, (95% CI 0.10–0.30) were detected in the direct‐mail group compared to 86 cases corresponding to 0.22% per invited woman (95% CI 0.20–0.30) and 0.64% per distributed HPV self‐sample kit (95% CI 0.50–0.80) in the opt‐in group. With respect to the relative cost for detection of ≥CIN2 and ≥CIN3 in the direct‐mail group compared to the opt‐in group, we found that 2.8 times more kits were mailed in the direct‐mail group to detect one case of ≥CIN2 and similarly 2.8 times more kits were mailed in the direct‐mail group to detect one case of ≥CIN3.

## 4 DISCUSSION

To assess participation and cost of two different HPV self‐sampling invitation strategies, a total of 49,393 screening non‐attenders were invited: 9639 with a direct‐mail invitation (intervention) and 39,754 with an opt‐in invitation (control). This randomized, pragmatic study was nested into the routine screening program of the Capital Region of Denmark. We found a significantly higher HPV self‐sampling participation by direct‐mail (25.2%) compared to opt‐in (20.2%), OR = 1.33 (95% CI 1.26–1.40, *p* < .0001) corresponding to an absolute participation difference of 5.0%. This is a lesser difference in participation between distribution methods than the 9.7% difference found in the recent meta‐analysis by Costa et al.,^16^ yet within the ranges of findings synthesized in meta‐analysis. Studies comparable to our design from Norway^19^ and Denmark^28^ show differences in absolute HPV self‐sampling participation by direct‐mail vs. opt‐in of 10.5% and 11.1%, respectively. In our study, we observe a higher absolute HPV self‐sampling participation in both groups than most studies^16^ and we have routinely reported an opt‐in participation in screening non‐attenders of 13%–20%^20^, ^27^, ^30^ with more than 200,000 women invited the past 10 years. Target population and local operational settings may drive this high opt‐in participation. HPV self‐sampling as a screening option in our setting has been developed over 10 years and has reached a level of operational maturity. To facilitate easy opt‐in, the woman can access a custom‐designed, dedicated website optimized to all electronic platforms, which is straightforward and convenient to use. The linkage between the woman and the unique identifier of the HPV self‐sampling kit requires no paperwork on the part of the woman, making it simple and straightforward to participate. The difference in participation between the direct‐mail and opt‐in group narrows with age and we found no difference in HPV self‐sampling participation according to invitation strategy in women above 50 years. A plausible interpretation could be that participation among non‐responder women older than 50 years is an active choice based on awareness raised by information material online and in the mailed HPV self‐sample kit more than the simple convenience of being presented with the kit straight in the mailbox. We also found that even though the direct‐mail approach recruits more women, participation among long‐term unscreened women was similar for the two invitation strategies. Looking at the ITS participation, including the clinician collected samples registered after invitation to HPV self‐sampling, the screening participation increased similar in the two groups by 10.1% in the direct‐mail group, and 10.9% in the opt‐in group, resulting in an overall participation of 35.3% and 31.1%, respectively. The 10%–11% addition to screening participation by clinician collected samples is on par with our previous experiences, and thus not considered study specific.^20^ For return‐of‐sample timespan, most HPV self‐samples were performed and sent to the laboratory for analysis shortly after receiving the HPV self‐sample kit or immediately after receiving the reminder to return the sample. No difference in the median time from shipment of HPV self‐sample kit to return of sample to laboratory were observed between groups. We found a seemingly higher initial return rate in the opt‐in group; it could be reasoned that woman making an active choice to participate are motivated to return the HPV self‐sample for analysis in a timely fashion. The return‐of‐sample analysis underlines that an active reminder policy to women who have not returned the HPV self‐sample after a defined period is appropriate and efficient. The overall HPV prevalence in the HPV self‐samples was similar between the groups; 14.6% (95% CI 13.2–16.0) for direct‐mail and 14.6% (95% CI 13.9–15.4) for opt‐in across all age groups (adjusted OR = 0.96, 95% CI 0.85–1.10). This is in line with our previous study reporting an overall HPV prevalence of 15.4% (95% CI 14.7–16.2).^20^ Not surprisingly, the youngest women had the highest prevalence, decreasing with increased age (*p* < .0001). A smaller Danish study by Tranberg et al.^28^ reported a slightly lower positive rate of 13.0% (95% CI 10.9–15.4) in a 30–64 year old cohort, however the difference could stem from the different age distribution as well as choice of Cobas 4800 HPV test platform, whereas we use the BD Onclarity HPV test in an optimized HPV self‐sample test protocol developed over many years.^31^ Adherence to recommended follow‐up after HPV self‐sampling is critical and lower adherence to clinician collected triage testing can challenge the effectiveness of HPV self‐sampling.^32^ In their meta‐analysis of 20 international trials, Arbyn et al. estimated 80.6% adherence to recommended follow‐up.^17^ Here, we observe that adherence to recommended follow‐up was similar between the groups and without age‐specific differences; 86.4% and 89.6%, respectively, which corresponds to previously published Danish data.^20^, ^28^ Again, we speculate that the logistic set‐up with automated letters delivered to the woman with a reminder to attend follow‐up at the clinician, assists to accomplish high follow‐up rates. In terms of detection of disease, the approaches were equally good. When adjusting for age and screening history, no difference between direct‐mail and opt‐in for detection of ≥CIN2 (adjusted OR = 0.84, 95% CI 0.57–1.23) or ≥CIN3 (adjusted OR = 0.86, 95% CI 0.54–1.38) were found. On a general note, we find that women above the age of 50 years had lower risk of disease compared to women below the age of 50 years, as well as long‐term unscreened women (≥10 years) having a higher risk of previously undetected disease. To compare the cost of the two invitation strategies, we calculated the difference in participation between groups and the relative cost of additional participants in terms of HPV self‐sample kits. The calculation showed that every additional woman screened in the direct‐mail group compared to the opt‐in group required 14 HPV self‐sampling kits distributed. Put in operational terms, the difference amounts to approximately 13,000 mailed kits never returned for 1000 additional participants in the direct‐mail group. High‐grade CIN detection rates are similar between invitation strategies, and the number of cases detected will thus be a direct function of the number of participants. Direct‐mail required 295 distributed kits and 74 received, valid HPV self‐samples per detected ≥CIN2 whereas opt‐in required 105 distributed kits and 62 received, valid HPV self‐samples per detected ≥CIN2. The additional cost of a direct‐mail strategy could be acceptable, if the cost difference can be offset by the reduced administrative costs of direct‐mail compared to the operation of an opt‐in program. Furthermore, this study has a limitation by not including information on socioeconomic status such as income, occupation, civil status, ethnicity, and education. By including such factors, more explicit insights into which target groups could benefit from a direct‐mail approach in terms of gaining participation would be instrumental. However, the randomized study design secured an equal distribution of unknown confounders and the pragmatic design of the study, which was embedded into the routine screening program covering the full non‐attender screening population, is regarded as a strength given that it maximizes the external validity of the study.

## 5 CONCLUSIONS

A direct‐mail invitation for HPV self‐sampling yielded a significantly higher participation than an opt‐in invitation when targeting screening non‐attenders. Participation increased by age for both strategies and decreased the longer the time since last screening. Short‐term unscreened women were more likely to return an HPV self‐sample by direct‐mail than opt‐in, however among long‐term unscreened women participation were similar for the two strategies. Adherence to follow‐up after a positive HPV self‐sample was high, approximating 90% independent of invitation strategy. Detection of ≥CIN2 and ≥CIN3 was similar between invitation strategies underlining that the major benefit of direct‐mail is solely the additional number of participants generated. The cost of each additional participant by direct‐mail compared to opt‐in equaled to 14 distributed kits.

## AUTHOR CONTRIBUTIONS

**Birgitte Tønnes Pedersen:** Data curation; formal analysis; methodology; validation; writing – original draft; writing – review and editing. **Si Brask Sonne:** Data curation; formal analysis; investigation; validation; writing – original draft. **Helle Pedersen:** Investigation; methodology; validation; writing – original draft. **Emilie Korsgaard Andreasen:** Data curation; investigation; validation; writing – original draft; writing – review and editing. **Reza Serizawa:** Project administration; resources; supervision; writing – original draft. **Ditte Møller Ejegod:** Conceptualization; funding acquisition; investigation; project administration; writing – original draft. **Jesper Bonde:** Conceptualization; formal analysis; funding acquisition; investigation; methodology; project administration; resources; supervision; validation; visualization; writing – original draft; writing – review and editing.

## CONFLICT OF INTEREST STATEMENT

JB reports being appointed member of Danish Steering Committee of Cervical Cancer screening has received reagents ad funding for studies to institution from Self‐screen (NL), Biocartis (B), has received honorarium for lectures from BD Integrated Diagnostic Systems (US), and honorarium for advisory board activity from Roche Molecular Systems, and MSD. RS report consultancy for Merck. The other authors report no conflict of interest.

## ETHICS STATEMENT

The HPV self‐sampling offer is an integral part of the cervical screening program in the Capital Region of Denmark. Extending the offer by a direct‐mail invitation is a temporary service change within the context of a quality development activity and considered as an extended service for the women allocated to this group. The study does not require medical ethical committee approval as per Danish Health Act §41, section 2. The study was conducted under the Capital Region of Denmark appropriation for HPV self‐sampling.

## Supporting information


Data S1.

## Data Availability

The data that support the findings of this study will be made available from the corresponding author in an aggregated format on request and pending approval from the Danish Health Data Authority (if required).

## References

[1] Arbyn M , Weiderpass E , Bruni L , et al. Estimates of incidence and mortality of cervical cancer in 2018: a worldwide analysis. Lancet Glob Health. 2020;8(2):e191‐e203. 31812369 10.1016/S2214-109X(19)30482-6 PMC7025157

[2] Bonde J , Bottari F , Iacobone AD , et al. Human papillomavirus same genotype persistence and risk: a systematic review. J Low Genit Tract Dis. 2021;25(1):27‐37. 33105450 10.1097/LGT.0000000000000573 PMC7748037

[3] Schiffman M , Castle PE , Jeronimo J , Rodriguez AC , Wacholder S . Human papillomavirus and cervical cancer. Lancet. 2007;370(9590):890‐907. 17826171 10.1016/S0140-6736(07)61416-0

[4] Vaccarella S , Lortet‐Tieulent J , Plummer M , Franceschi S , Bray F . Worldwide trends in cervical cancer incidence: impact of screening against changes in disease risk factors. Eur J Cancer. 2013;49(15):3262‐3273. 23751569 10.1016/j.ejca.2013.04.024

[5] World Health Organization . Global Strategy to Accelerate the Elimination of Cervical Cancer as a Public Health Problem. World Health Organization; 2020.

[6] Wang J , Elfstrom KM , Andrae B , et al. Cervical cancer case‐control audit: results from routine evaluation of a nationwide cervical screening program. Int J Cancer. 2020;146(5):1230‐1240. 31107987 10.1002/ijc.32416 PMC7003887

[7] Dugue PA , Lynge E , Bjerregaard B , Rebolj M . Non‐participation in screening: the case of cervical cancer in Denmark. Prev Med. 2012;54(3–4):266‐269. 22300964 10.1016/j.ypmed.2012.01.012

[8] RKKP . Årsrapport 2023 – Dansk Kvalitetsdatabase for Livmoderhalskræftscreening. 2024.

[9] Bruni L , Serrano B , Roura E , et al. Cervical cancer screening programmes and age‐specific coverage estimates for 202 countries and territories worldwide: a review and synthetic analysis. Lancet Glob Health. 2022;10(8):e1115‐e1127. 35839811 10.1016/S2214-109X(22)00241-8 PMC9296658

[10] Chorley AJ , Marlow LA , Forster AS , Haddrell JB , Waller J . Experiences of cervical screening and barriers to participation in the context of an organised programme: a systematic review and thematic synthesis. Psychooncology. 2017;26(2):161‐172. 27072589 10.1002/pon.4126 PMC5324630

[11] Harder E , Thomsen LT , Hertzum‐Larsen R , et al. Determinants for participation in human papillomavirus self‐sampling among nonattenders to cervical cancer screening in Denmark. Cancer Epidemiol Biomarkers Prev. 2018;27(11):1342‐1351. 30108095 10.1158/1055-9965.EPI-18-0480

[12] Leinonen MK , Campbell S , Ursin G , Trope A , Nygard M . Barriers to cervical cancer screening faced by immigrants: a registry‐based study of 1.4 million women in Norway. Eur J Public Health. 2017;27(5):873‐879. 28957477 10.1093/eurpub/ckx093 PMC5881680

[13] Basu P , Ponti A , Anttila A , et al. Status of implementation and organization of cancer screening in the European Union member states‐summary results from the second European screening report. Int J Cancer. 2018;142(1):44‐56. 28940326 10.1002/ijc.31043

[14] Ronco G , Dillner J , Elfstrom KM , et al. Efficacy of HPV‐based screening for prevention of invasive cervical cancer: follow‐up of four European randomised controlled trials. Lancet. 2014;383(9916):524‐532. 24192252 10.1016/S0140-6736(13)62218-7

[15] Arbyn M , Ronco G , Anttila A , et al. Evidence regarding human papillomavirus testing in secondary prevention of cervical cancer. Vaccine. 2012;30(Suppl 5):F88‐F99. 23199969 10.1016/j.vaccine.2012.06.095

[16] Costa S , Verberckmoes B , Castle PE , Arbyn M . Offering HPV self‐sampling kits: an updated meta‐analysis of the effectiveness of strategies to increase participation in cervical cancer screening. Br J Cancer. 2023;128(5):805‐813. 36517552 10.1038/s41416-022-02094-w PMC9977737

[17] Arbyn M , Smith SB , Temin S , et al. Detecting cervical precancer and reaching underscreened women by using HPV testing on self samples: updated meta‐analyses. BMJ. 2018;363:k4823. 30518635 10.1136/bmj.k4823 PMC6278587

[18] Elfstrom KM , Sundstrom K , Andersson S , et al. Increasing participation in cervical screening by targeting long‐term nonattenders: randomized health services study. Int J Cancer. 2019;145(11):3033‐3039. 31032904 10.1002/ijc.32374

[19] Aasbo G , Trope A , Nygard M , et al. HPV self‐sampling among long‐term non‐attenders to cervical cancer screening in Norway: a pragmatic randomised controlled trial. Br J Cancer. 2022;127(10):1816‐1826. 35995936 10.1038/s41416-022-01954-9 PMC9643532

[20] Ejegod DM , Pedersen H , Pedersen BT , Serizawa R , Bonde J . Operational experiences from the general implementation of HPV self‐sampling to Danish screening non‐attenders. Prev Med. 2022;160:107096. 35594924 10.1016/j.ypmed.2022.107096

[21] Sultana F , Gertig DM , English DR , et al. HPV self‐sampling and follow‐up over two rounds of cervical screening in Australia ‐ the iPap trial. J Med Screen. 2022;29(3):185‐193. 35313763 10.1177/09691413221080635

[22] Tranberg M , Petersen LK , Hammer A , et al. Value of a catch‐up HPV test in women aged 65 and above: a Danish population‐based nonrandomized intervention study. PLoS Med. 2023;20(7):e1004253. 37410699 10.1371/journal.pmed.1004253 PMC10325045

[23] Silveira MFD , Buffarini R , Gaspar PC , et al. Detection of HPV DNA in vaginal samples self‐collected by women living with HIV treated through the Brazilian public health system: prevalence and analysis of risk factors. Rev Soc Bras Med Trop. 2023;56:e02772023. 37820103 10.1590/0037-8682-0277-2023 PMC10561897

[24] Serrano B , Ibanez R , Robles C , Peremiquel‐Trillas P , de Sanjose S , Bruni L . Worldwide use of HPV self‐sampling for cervical cancer screening. Prev Med. 2022;154:106900. 34861338 10.1016/j.ypmed.2021.106900

[25] Lam JUH , Elfstrom KM , Ejegod DM , et al. High‐grade cervical intraepithelial neoplasia in human papillomavirus self‐sampling of screening non‐attenders. Br J Cancer. 2018;118(1):138‐144. 29136403 10.1038/bjc.2017.371 PMC5765223

[26] Lam JUH , Rebolj M , Ejegod DM , et al. Prevalence of human papillomavirus in self‐taken samples from screening nonattenders. J Clin Microbiol. 2017;55(10):2913‐2923. 28724554 10.1128/JCM.00550-17 PMC5625377

[27] Lam JU , Rebolj M , Moller Ejegod D , et al. Human papillomavirus self‐sampling for screening nonattenders: opt‐in pilot implementation with electronic communication platforms. Int J Cancer. 2017;140(10):2212‐2219. 28195317 10.1002/ijc.30647 PMC5516138

[28] Tranberg M , Bech BH , Blaakaer J , Jensen JS , Svanholm H , Andersen B . Preventing cervical cancer using HPV self‐sampling: direct mailing of test‐kits increases screening participation more than timely opt‐in procedures – a randomized controlled trial. BMC Cancer. 2018;18(1):273. 29523108 10.1186/s12885-018-4165-4 PMC5845195

[29] Zwarenstein M , Treweek S , Gagnier JJ , et al. Improving the reporting of pragmatic trials: an extension of the CONSORT statement. BMJ. 2008;337:a2390. 19001484 10.1136/bmj.a2390 PMC3266844

[30] Pedersen BT , Pedersen H , Serizawa R , Sonne SB , Andreasen EK , Bonde J . Cervical cancer screening activity in the Capital Region of Denmark before, during and after the COVID‐19 pandemic. Prev Med. 2024;180:107888. 38325609 10.1016/j.ypmed.2024.107888

[31] Martinelli M , Latsuzbaia A , Bonde J , et al. Performance of BD Onclarity HPV assay on FLOQSwabs vaginal self‐samples. Microbiol Spectr. 2024;12(3):e0287223. 38323823 10.1128/spectrum.02872-23 PMC10913526

[32] Rebolj M , Sargent A , Njor SH , Cuschieri K . Widening the offer of human papillomavirus self‐sampling to all women eligible for cervical screening: make haste slowly. Int J Cancer. 2023;153(1):8‐19. 36385698 10.1002/ijc.34358 PMC10952475

