# Supplementary material for: Participation and relative cost of attendance by direct‐mail compared to opt‐in invitation strategy for HPV self‐sampling targeting cervical screening non‐attenders: A large‐scale, randomized, pragmatic study
Source: Int J Cancer. 2024 Nov 23;156(8):1594–605. doi: 10.1002/ijc.35263 (PMC11826108; doi:10.1002/ijc.35263)
Supplement: Supplementary file 1 — Data S1. [file IJC-156-1594-s001.pdf]

Supplementary Figure

Participation and relative cost of attendance by direct-mail compared to opt-in invitation strategy for HPV self-sampling targeting cervical screening non-attenders: a large-scale, randomized, pragmatic study.

Birgitte Tønnes Pedersen, Si Brask Sonne, Helle Pedersen, Emilie Korsgaard Andreasen, Reza Serizawa, Ditte Møller Ejegod & Jesper Bonde

Table of content

Supplementary Figure 1: Algorithm for clinical management of HPV self-samples

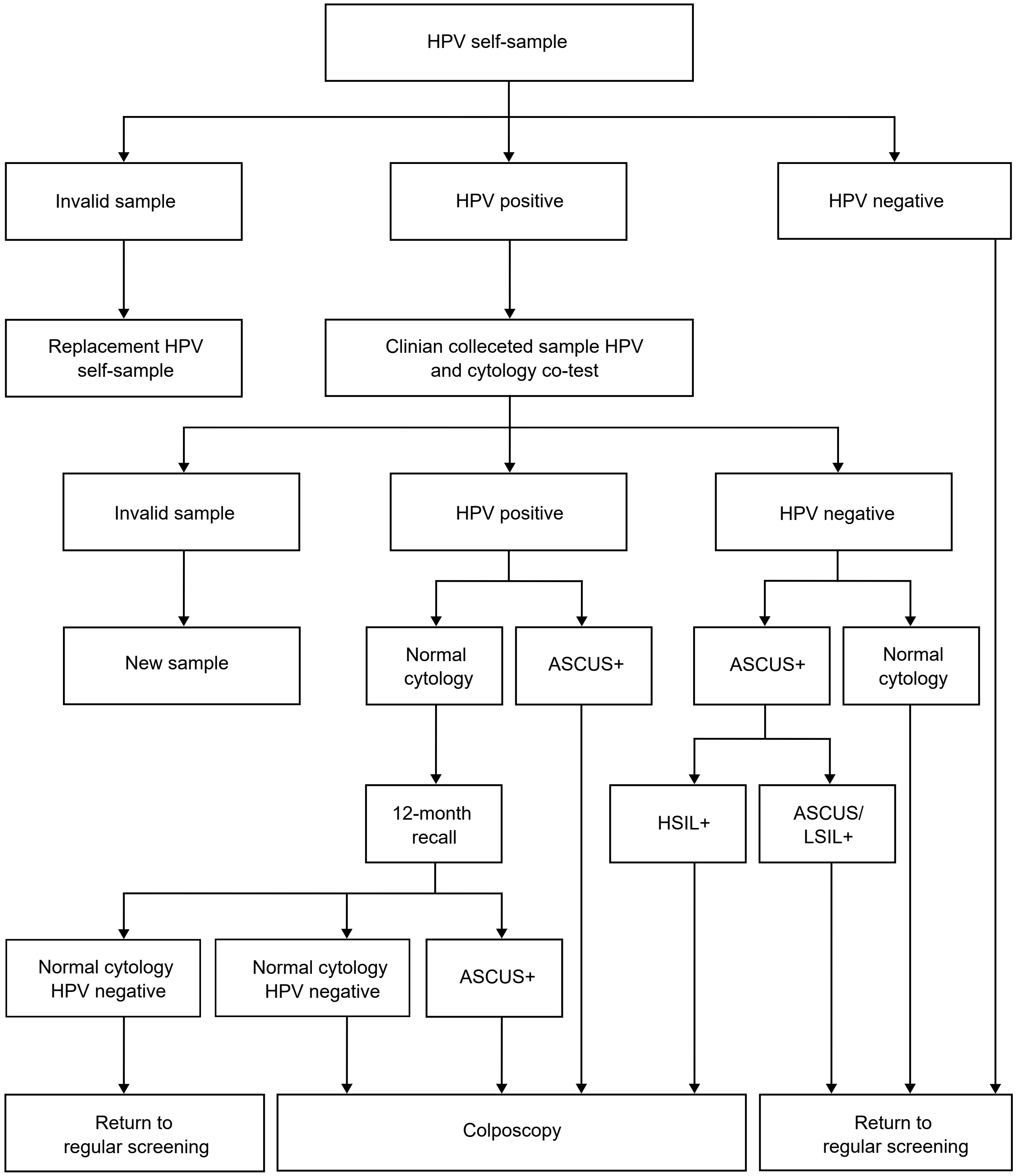

Supplementary Figure 1: Algorithm for clinical management of HPV self-samples
